# Identifying EGFR-Expressed Cells and Detecting EGFR Multi-Mutations at Single-Cell Level by Microfluidic Chip

**DOI:** 10.1007/s40820-017-0168-y

**Published:** 2017-11-14

**Authors:** Ren Li, Mingxing Zhou, Jine Li, Zihua Wang, Weikai Zhang, Chunyan Yue, Yan Ma, Hailin Peng, Zewen Wei, Zhiyuan Hu

**Affiliations:** 1 CAS Key Laboratory of Standardization and Measurement for Nanotechnology, CAS Key Laboratory for Biomedical Effects of Nanomaterials and Nanosafety, CAS Center for Excellence in Nanoscience, National Center for Nanoscience and Technology of China, Beijing, 100190 People’s Republic of China; 2 Academy for Advanced Interdisciplinary Studies, Peking University, Beijing, 100871 People’s Republic of China; 3 Sino-Danish College, University of Chinese Academy of Sciences, Beijing, 100049 People’s Republic of China; 4 Yangtze River Delta Academy of Nanotechnology and Industry Development Research, Jiaxing, 314000 Zhejiang Province People’s Republic of China; 5 University of Chinese Academy of Sciences, Beijing, 100049 People’s Republic of China

**Keywords:** EGFR mutation, Single-cell analysis, Microfluidic chip, Tyrosine kinase inhibitor

## Abstract

EGFR mutations companion diagnostics have been proved to be crucial for the efficacy of tyrosine kinase inhibitor targeted cancer therapies. To uncover multiple mutations occurred in minority of EGFR-mutated cells, which may be covered by the noises from majority of un-mutated cells, is currently becoming an urgent clinical requirement. Here we present the validation of a microfluidic-chip-based method for detecting EGFR multi-mutations at single-cell level. By trapping and immunofluorescently imaging single cells in specifically designed silicon microwells, the EGFR-expressed cells were easily identified. By in situ lysing single cells, the cell lysates of EGFR-expressed cells were retrieved without cross-contamination. Benefited from excluding the noise from cells without EGFR expression, the simple and cost-effective Sanger’s sequencing, but not the expensive deep sequencing of the whole cell population, was used to discover multi-mutations. We verified the new method with precisely discovering three most important EGFR drug-related mutations from a sample in which EGFR-mutated cells only account for a small percentage of whole cell population. The microfluidic chip is capable of discovering not only the existence of specific EGFR multi-mutations, but also other valuable single-cell-level information: on which specific cells the mutations occurred, or whether different mutations coexist on the same cells. This microfluidic chip constitutes a promising method to promote simple and cost-effective Sanger’s sequencing to be a routine test before performing targeted cancer therapy.

**Electronic supplementary material:**

The online version of this article (10.1007/s40820-017-0168-y) contains supplementary material, which is available to authorized users.

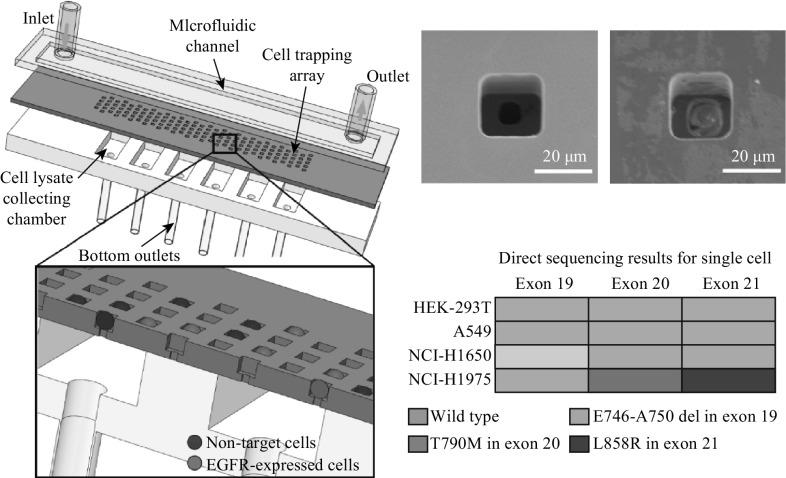

## Highlights

- Discovering not only the existence of specific EGFR multi-mutations occurred in minority of EGFR-mutated cells which may be covered by the noises from majority of un-mutated cells, but also other valuable single-cell-level information: on which specific cells the mutations occurred, or whether different mutations coexist on the same cells.
- Trapping and identifying EGFR-expressed single cells to exclude interferences from EGFR-unexpressed cells.

## Introduction

Epidermal growth factor receptor (EGFR) has been proved to be related with the pathogenesis and progression of multiple carcinoma types, including lung cancer [1], breast cancer [2], prostatic cancer [3] and pancreatic cancer [4]. Previous clinical trials demonstrated that inhibitors of EGFR tyrosine kinase (TK) effectively retarded disease progression of non-small cell lung cancer (NSCLC) patients [5, 6]. Evidences suggest that mutated EGFR proteins are inhibited by small-molecule tyrosine kinase inhibitors (TKIs) which compete with ATP binding to the TK domain of the receptor and block signal transduction [6]. Mutations mediate oncogenic effects by altering downstream signaling and anti-apoptotic mechanisms [1, 7]. For instance, L858R in exon 21 and Del E749-A750 in exon 19 mutations increase the TKIs sensitivity [8], while T790M in exon 20 is a drug-resistant mutation, abrogating inhibitors binding with EGFR [9, 10]. Since these mutations significantly affect the effectiveness of targeted medicine, EGFR analysis is becoming more and more a routine test before selecting targeted therapy for related cancers, such as NSCLC [11–13].

Immunohistochemistry of tumor tissue is the most clinically used method to detect EGFR at protein level [14, 15]. Also, directly sequencing cells extracted from tumor tissue has also been clinically accepted to detect EGFR mutation sequences [16, 17]. However, either the protein analysis or the gene sequencing of tumor tissue provides only averaged information of the whole cell population. Since the tumor cells are heterogeneous [18, 19], the mutations occurred on a small amount of cells could be covered by the other normal cells [20].

To reveal EGFR mutation on individual cells, fluorescence-activated cell sorting (FACS) was previously introduced [21] to sort single cells from a large cell amount, usually larger than 10^5^ cells [22]. For cell samples fewer than 10^5^ cells, the emerging microfabrication technologies have advanced the examinations of protein expression or gene mutation at single-cell level by preciously controlling single cells and their surrounding environments. At protein level, by employing immunofluorescence identification, microfluidic chips are capable of identifying [23, 24] or enumerating [25] EGFR-expressed cells. However, the application of protein level analyses is limited by the diverse specificity of different antibodies and the lack of detailed mutation information. At gene level, on-chip single-cell isolation, lysis and gene amplification have been realized using microchambers [26] or droplets [27], enabling the sequencing of the disease-related gene fragments [28, 29] or even the whole genome [30]. However, the lack of on-chip identification of EGFR expression and corresponding sorting of EGFR-expressed cells compromises the feasibility of selectively sequencing EGFR-expressed cells which possibly make up a small portion of all cells extracted from tumor tissue.

Clinically, before performing targeted therapy, it is crucial to understand not only if EGFR expression happens but also how many types of disease-related mutation exist and what the mutated sequences exactly are [31]. This urgent demand is yet to be fulfilled with an accurate, simple and cost-effective method, despite the advances which have already been achieved on EGFR mutation determination, with or without the assistance of microfluidic chips. To address this requirement, we developed a simple microfluidic chip to simultaneously finish on-chip cell identification and in situ cell lysis for detecting EGFR multi-mutations at single-cell level. The on-chip cell identification distinguished EGFR-expressed cells from EGFR-unexpressed cells, providing direct and accurate information about the portion of EGFR-expressed cells. Also, by sequencing only EGFR-expressed cells, the interference from EGFR-unexpressed cells was excluded. The in situ cell lysis ensured the accuracy of DNA sequence by avoiding cross-contamination between different cells and possible cell loss while transferring cells between on-chip and off-chip. After optimizing the operation of the microfluidic chip, we evaluated its performance with NSCLC cells. The results demonstrated that the microchip accurately distinguished NSCLC cells from normal cells and determined three important drug-related EGFR mutations that the NSCLC cells possessed.

## Experimental Section

### Materials and Cells

Dulbecco’s modified eagle’s medium (DMEM), fetal bovine serum (FBS), penicillin–streptomycin and trypsin were purchased from Life Technologies, USA. Phosphate-buffered saline (PBS, pH 7.4) was purchased from Sigma-Aldrich, USA. EGFR monoclonal antibodies conjugated with fluorescein isothiocyanate (anti-EGFR-FITC) and epithelial cell adhesion molecule monoclonal antibodies conjugated with fluorescein isothiocyanate (anti-EpCAM-FITC) were both purchased from Abcam, USA. The nuclear dye, 4′,6-diamidino-2-phenylindole (DAPI), was purchased from Sigma-Aldrich, USA. Silicon wafers were purchased from Xilika, China. Polydimethylsiloxane (PDMS) was obtained from Dow Corning, USA. Multiple displacement amplification (MDA) REPLI-g single-cell kits were purchased from Qiagen, Germany. Cell lysis buffer and polymerase chain reaction (PCR) kits were purchased from Tiangen, China.

The non-small cell lung cancer cell line NCI-H1975 and NCI-H1650 were cultured in 1640 medium with 1% penicillin–streptomycin and 10% fetal bovine serum (FBS). Non-small cell lung cancer cell line A549, breast cancer cell line MCF-7 and human embryonic kidney cell line HEK-293T were cultured in DMEM medium with 1% penicillin–streptomycin and 10% FBS. All cells were incubated at 37 °C under 5% CO_2_ atmosphere. Before experiments, cells were fixed using a 4% paraformaldehyde solution and then labeled by immunofluorescence. All cell lines were stained by DAPI to indicate cell nuclei. MCF-7 and HEK-293T were mixed and stained by Anti-EpCAM-FITC. A549, NCI-H1975, NCI-H1650, and HEK-293T were mixed and stained by anti-EGFR-FITC. Then cells were rinsed three times to exclude excessive fluorescently labeled antibodies.

### Fluorescently Identifying, In Situ Lysing, Amplifying and Directly Sequencing MCF-7 Cells

MCF-7 and HEK-293 cells were mixed at a cell number ratio of 1:10 in a tube. All cells were treated with DAPI and anti-EpCAM-FITC staining. Then the cell concentration was regulated to 3.2 × 10^5^ Cells mL^−1^. The cell mixture was pumped in the chip at a flow rate of 3 μL min^−1^ for 1 min from the inlet by a syringe pump, followed by pausing the flow for 3 min till cells were trapped in microwells. The pumping–pausing procedure was repeated for three times. After rinsing the chip by PBS with a flow rate of 30 μL min^−1^, all cells on chip were fluorescently imaged by a confocal microscope (Zeiss 710, Zeiss, Germany) with an automatic stepper stage. All images were manually checked to select chambers which contained only EGFR-expressed cells. The imaging and cell selecting costed 30 min. After that, lysis buffer was pumped into the chip from the inlet by a syringe pump. Then the chip was placed at 4 °C for 30 min to lyse all cells. The cell lysates from selected chambers were retrieved into PCR tubes and amplified, respectively. Following the instruction of REPLI-g single-cell MDA kits, the cell lysate was amplified at 30 °C for 3 h by MDA reaction. The amplification product was diluted by double distilled water (100 times dilution) and transferred to another tube (2 μL per tube). Then 15 μL PCR mixture, 12 μL double distilled water, 0.5 μL forward prime and 0.5 μL reverse prime were added in the tube for a standard PCR to amplify STR (short tandem repeat) domain sequence. PCR cycling conditions were as follows: 94 °C for 5 min, 35 cycles (30 s per cycle) of 94 °C, 60 °C for 30 s, 72 °C for 45 s, 1 cycle of 72 °C for 10 min and maintain at 4 °C. The primers are:

- Forward primer: 5′-TCTAGCAGCAGCTCATGGTG-3′;
- Reverse primer: 5′-GGAGCCCAAGGTTCTGAGT-3′.

The PCR was finished in 1.5 h. 5 μL amplification products were verified by fluorescently imaging of agarose gel. Then the rest of 25 μL amplification products were sent for sequencing (Ruibo, Beijing), which was finished in 24 h.

### Detecting EGFR Multi-Mutations

Processing EGFR-mutated cells shares most the protocols of processing MCF-7 cells. The differences are the following: (1) staining all cells with anti-EGFR-FITC and DAPI; (2) the A549, NCI-H1975, NCI-H1650, and HEK-293T cells were mixed at a ratio of 1:1:1:15; (3) using different PCR primers to amplify different domains,

- Exon 19 forward: 5′-AACGTCTTCCTTCTCTCTCTGTCAT-3′
- Exon 19 reverse: 5′-CACACAGCAAAGCAGAAACTCAC-3′
- Exon 20 forward: 5′-ACCATGCGAAGCCACACTGACGTGCCTCTCCCTCCCTCCAG-3′
- Exon 20 reverse: 5′-GTAATCAGGGAAGGGAGATACGGGGAGGGGAGATAAGGAGCCA-3′
- Exon 21 forward: 5′-CCCTCACAGCAGGGTCTT-3′
- Exon 21 reverse: 5′-GTCTGACCTAAAGCCACCTC-3′

## Results and Discussion

From the clinical point of view, an ideal technology for detecting EGFR multi-mutations should achieve the following: (1) accurate enough to precisely provide sequence information about specific kinds of mutations; (2) simple and cost-effective to be accepted as a routine test before cancer targeted therapy. To fulfill these requirements, we developed a microwells array-based microfluidic chip to firstly identify EGFR-expressed cells from EGFR-unexpressed cells, then in situ lysis all EGFR-expressed cells for the following gene sequencing.

### The Microfluidic Chip

**Fig. 1:**
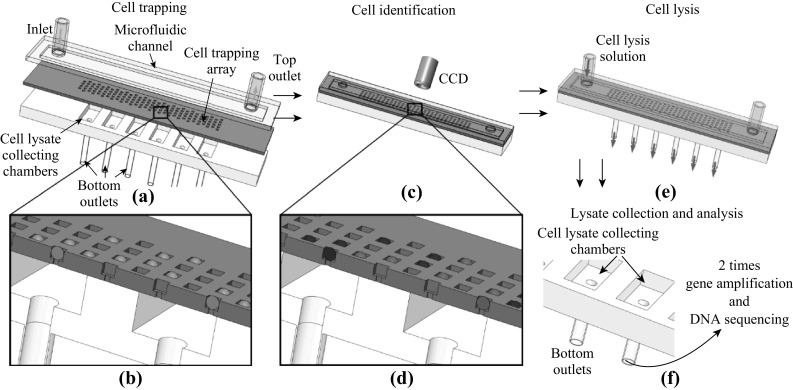
The schematic view of microfluidic chip and its operation. **a** The cell mixture is pumped into a microfluidic chip which consists of three layers: the microfluidic channel, the cell trapping array and the cell lysate collecting chamber. The flow velocity was 3 μL min^−1^. **b** Cells are trapped in microwells. The square microwell is designed to fit only one cell. **c** The chip was fluorescently examined to identify cells with specific protein expression. **d** Blue balls represent negative cells which exhibit only blue color of DAPI, while green balls represent positive cells which exhibit both green color of FITC and blue color of DAPI. **e** The cells in microwells are lysed by inputting cell lysis solution through the microfluidic channel. The cell lysate is directed to cell lysate collecting chambers. **f** The cell lysates are separately collected from cell lysate collecting chambers for the following DNA amplification and sequencing. To clearly demonstrate the chip structure, the schematic figures do not follow the exact well number and dimension

As shown in Fig. 1a, from the functional point of view, the microfluidic chip consisted of three layers: the microfluidic channel, the cell trapping array and the cell lysate collecting chambers. To perform EGFR multi-mutations analysis, the cell mixture, which may contain a small portion of EGFR-expressed cells and many other EGFR-unexpressed cells, was firstly incubated with DAPI and anti-EGFR-FITC, then pumped into the microfluidic channel (40 mm in length, 8 mm in width and 30 μm in height). The bottom outlets were closed while pumping cell mixture. When the channel was fully filled by cell mixture, the flow was paused till the majority of cells were captured by the cell trapping array (schemed in Fig. 1b). There were 30,000 square microwells in the cell trapping array. All wells were 25 µm in side length and 30 µm in depth. The whole cell trapping array was fluorescently imaged (schemed in Fig. 1c). The EGFR-expressed cells would be recognized by anti-EGFR-FITC and exhibit green fluorescence. Meanwhile, all cells would exhibit blue fluorescence of DAPI staining (schemed in Fig. 1d).

By analyzing fluorescent images, all EGFR-expressed cells were identified from EGFR-unexpressed and their positions were marked. To lyse all trapped cells, after opening all bottom outlets and closing top outlet, cell lysis solution was pumped into the channel to fill cell trapping array and all cell lysate collecting chambers (schemed in Fig. 1e). Then the bottom outlets were switched off for 30 min until all cells were fully lysed. The cell lysates were maintained in cell lysate collecting chambers through the through-hole at the bottom of each microwell. All through-holes were 8 µm in side length and 170 µm in depth. The through-hole design ensured (as simulated in Fig. S1) all cell lysates were transferred to collecting chamber, without any cross-contamination among different trapping wells.

Finally, the top outlet and all bottom outlets were opened, and cell lysates were retrieved through the bottom outlets, with the assistance of negative pressure, which was generated by an external syringe pump. The square cell lysate collecting chamber (1.5 mm in side length, 1 mm in depth and 2.25 µL in volume) was specially designed to be much larger than the cell trapping chamber. Each lysate collecting chamber covered 100 cell trapping chambers. By controlling the initial cell density, we realized that each lysate collecting chamber contains cell lysates from a few cells (< 4). As long as the ratio between EGFR-mutated and normal cells was more than 1:3, the mutated sequence could be detected by the Sanger’s sequencing method [32]. It meant that we could sequence all cells (< 4) from a chamber, which contained at least 1 EGFR-expressed cell, to detect if any specific mutation exists in EGFR-expressed cells.

In addition, it was easier to retrieve cell lysate from a larger chamber, avoiding the loss of cell lysate and corresponding inaccurate sequencing results. Multiple displacement amplification (MDA) was introduced for unbiased amplification of the whole genome of cell lysates. Depending on how many mutation types needed to be determined, the amplification product was divided into several parts which were, respectively, amplified again by polymerase chain reaction (PCR) with different primers for specific domains. The final amplification products were directly sequenced to reveal specific gene mutations (schemed in Fig. 1f). Compared with previous one-time PCR amplification [33] in which only one domain could be examined from lysates retrieved from one cell, the combination of MDA and PCR provided the capability of accurately sequencing different domains from the same cell lysate. On the other hand, for the aim of finding out if specific gene mutations exist in EGFR-expressed cells, not accurately sequencing the whole genome of every cell, our design is a practical alternative to expensive deep sequencing of single cells.

**Fig. 2:**
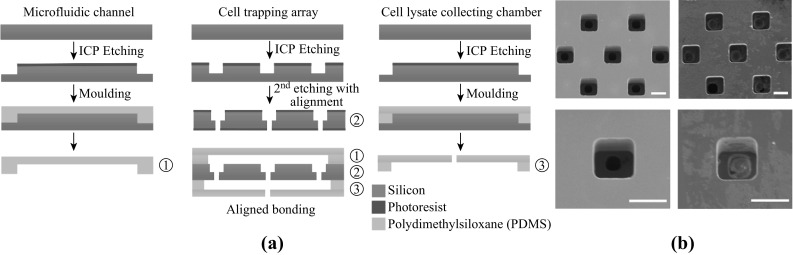
The fabrication of microfluidic chip. **a** The fabrication process of the microfluidic chip which consists of three layers. They are indicated by a number 1, 2, and 3, respectively. **b** SEM images of empty silicon microwells (left column) and microwells occupied by single cells (right column). Scale bar: 20 µm

Figure 2a shows the fabrication of the microfluidic chip. Both the microfluidic channel and cell lysate collecting chamber were fabricated by PDMS. By utilizing dry-etched 4-inch silicon wafer as the mold, the PDMS was molded to required structures. For cell lysate collecting chambers, through-holes were fabricated by piercing the PDMS layer (with needles) to form the lysate outlets. Silicon was used as the material for the cell trapping array, because the microwell and through-hole required precise dimensions and high width/depth ratio (1:17) which could only be fulfilled by silicon-based dry-etching. By etching a 200-µm-thick 4 inch silicon wafer from both sides with alignment, the 30-µm-deep microwells and the 170-µm-deep through-wholes were formed. Finally, after being treated with oxygen plasma for 60 s, two PDMS layers were bonded on both sides of the silicon wafer with alignment. Figure 2b shows the SEM images of microwells and through-holes. The cells captured in microwells are also imaged in Fig. 2b.

**Fig. 3:**
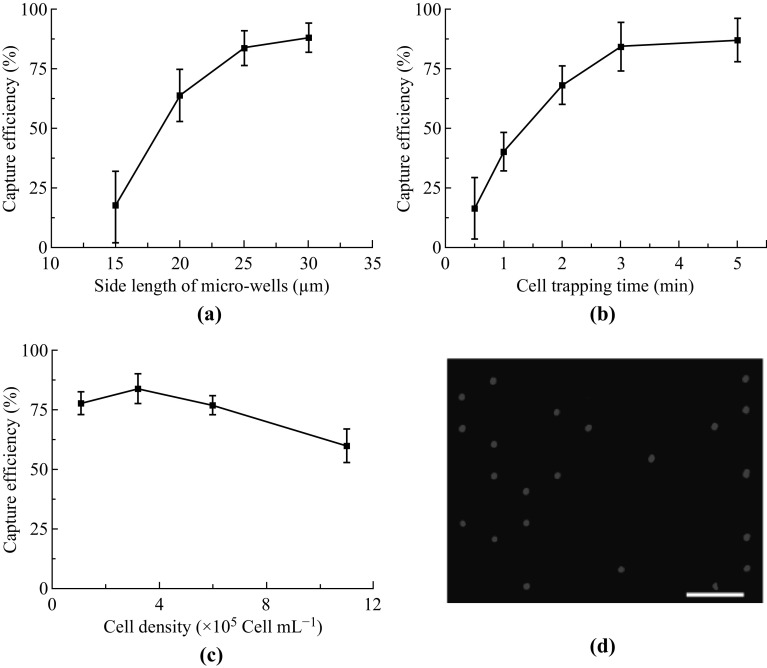
The optimization of cell capture efficiency. The cell capture efficiency was defined as the ratio between captured cells and all cells pumped into the microfluidic chip. **a** The relationship between cell capture efficiency and side length of square microwells. Other operation parameters are: 3.2 × 10^5^ Cells mL^−1^ and 3 min trapping time. **b** The relationship between cell capture efficiency and cell trapping time. Other operation parameters are: 25 µm in side lengths, and 3.2 × 10^5^ Cells mL^−1^. **c** The relationship between cell capture efficiency and cell density. Other operation parameters are: 25 µm side lengths and 3 min trapping time. In **a**, **b** and **c**, data are expressed as the mean ± SD from 3 independent assays. **d** Trapped single cells were stained by DAPI, which are blue spots. Scale bar: 100 µm

An efficient single-cell capture in microwells is crucial for fluorescent identification. Therefore, we evaluated the relationship between cell capture efficiency, the microwell size, the cell trapping time and the initial cell density. For all assays, the cell capture efficiency was defined as the ratio between captured cells and all cells pumped into the microfluidic chip. For all microwells-based cell trapping, the dimension of microwells was the key factor for single-cell trapping. We tested four different side lengths of square microwells, 15, 20, 25, and 30 µm. We used A549 cells for the evaluation. The cell density and trapping time were fixed at 3.2 × 10^5^ Cells mL^−1^ and 3 min, respectively. As shown in Fig. 3a, larger well dimension brought better capture efficiency. However, while utilizing 30 µm as well size, about 10% wells were occupied by two or more cells (Fig. S2). The cell overlapping would compromise the accuracy of fluorescent identification. Therefore, we selected 25 µm as the well side length to maximize the capture efficiency and avoid cell overlapping. We then evaluated the influence of different cell trapping times which were defined as the flow pausing time for settling single cells into microwells. The capture efficiencies were remarkably enhanced from 10% to about 80%, while the cell trapping times were increased from 0.5 to 3 min. Since further increasing cell trapping time did not improve capture efficiency as well, we used 3 min as the cell trapping time. We finally evaluated the influences of cell density. Four cell densities (1.1 × 10^5^, 3.2 × 10^5^, 6.0 × 10^5^ and 1.1 × 10^6^ Cells mL^−1^) were tested. As expected, the optimum capture efficiency (about 85%) occurred when we used a low cell density (3.2 × 10^5^ Cells mL^−1^). Overall, we used 25 µm for side length of all microwells, 3.2 × 10^5^ Cells mL^−1^ for cell density and 3 min for cell trapping time per cycle to realize 85% capture efficiency, which is enough for EGFR mutation analysis.

### Single-Cell Identification and DNA Sequencing for Detecting EGFR Multi-Mutation

**Fig. 4:**
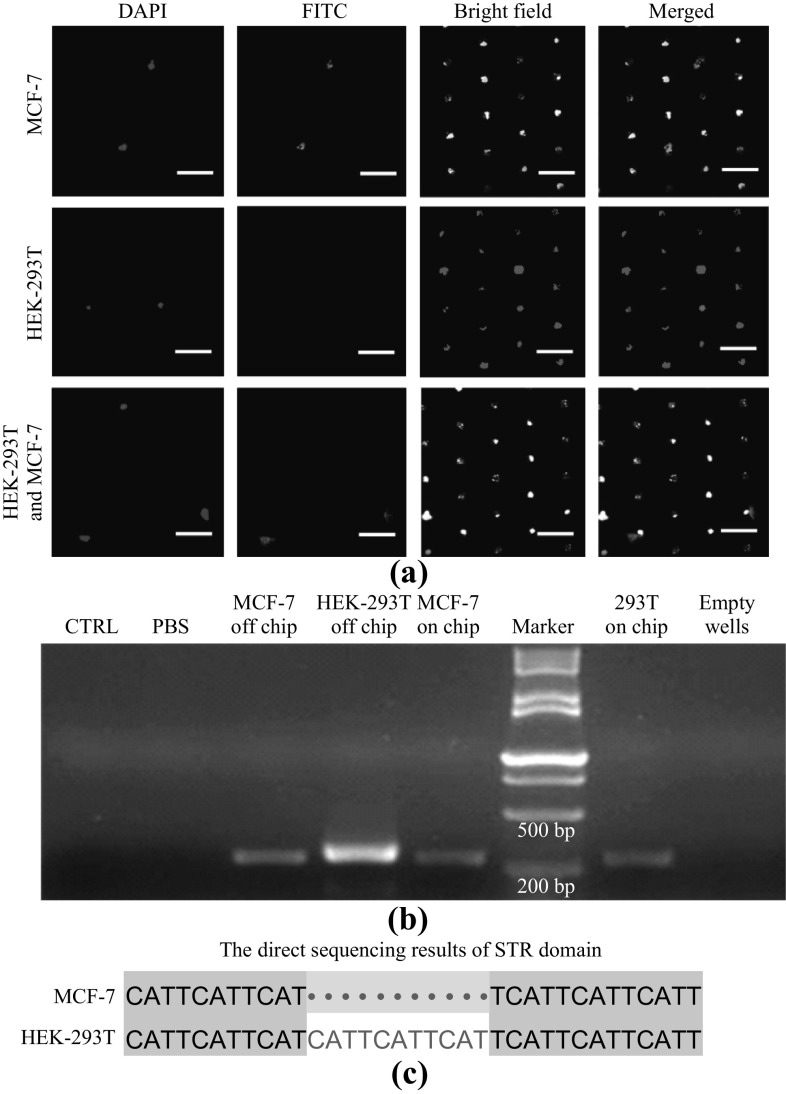
Fluorescently identifying, amplifying and sequencing MCF-7 cells. **a** The fluorescent images of cells in microwells. Upper row is an area contains only MCF-7 cells which exhibit both DAPI (blue) and FITC (green) staining; Middle row contains only HEK-293T cells which exhibit only DAPI (blue) staining; Lower row contains both HEK-293T cells and MCF-7 cells. Scale bar: 50 µm. **b** DNA amplification products of STR domain were verified by fluorescence image of agarose gel. Left two columns are the empty run and PBS reaction, both as negative control; The third and fourth left columns are the products from pure HEK-293T and MCF-7 cells, without the chip processing, both as positive control. Three right columns are products from chip areas contain no cells, only HEK-293T cells and only MCF-7 cells, sequentially. **c** The sequencing results for STR domain from the cells lysates from areas, respectively, contain only HEK-293T and MCF-7 cells. (Color figure online)

To evaluate the performance of the microfluidic chip on identifying and lysing targeted cells without cross-contamination, we mixed MCF-7 cells and HEK-293T cells at a ratio of 1:10. Detailed protocols are described in experimental section. Figure 4a shows the fluorescent images of captured cells in microwells. The blue spots indicate the DAPI-stained cell nuclei, while the green spots indicate MCF-7 cells expressing epithelial cell adhesion molecule (EpCAM) which is recognized by anti-EpCAM-FITC. The upper and middle rows show areas contain only MCF-7 and HEK-293T cells, respectively. While the lower row shows the area contains both MCF-7 and HEK-293T cells. After fluorescently identifying MCF-7 and HEK-293T cells, we sequenced their short tandem repeat (STR) domain to further confirm the identification results and to tell if cross-contaminations happened in cell lysing and gene amplification procedures. STR is a 2-6 bases short tandem repeat structure in gene sequence. Every cell line has its unique STR sequence. Therefore, sequencing STR gene fragment was wildly employed to identify specific cell types [34]. All cells in microwells were lysed in situ, and the cell lysates were, respectively, collected from related lysate collecting chambers. Using MDA, we amplified two kinds of cell lysates: (1) the lysate retrieved from areas which contain only MCF-7 cells; (2) the lysate retrieved from areas which contain only HEK-293T cells. The contents from those chambers which contain no cells were also treated by the same MDA procedure for experimental control. We then secondarily amplified all samples using PCR with primer designed for STR domains. Figure 4b shows correct PCR products from both MCF-7 and HEK-293T cell lysates. Meanwhile, no PCR product was detected in liquids collected from chambers which contained no cells, which indicated that no cross-contamination occurred between chambers occupied and unoccupied by cells. The sequencing results (Fig. 4c) further reveals that the quality of PCR products satisfies the requirement of Sanger’s sequencing, in addition, no cross-contamination occurred between chambers, respectively, occupied by MCF-7 and HEK-293T cells.

After verifying the feasibility of in situ identifying and lysing few cells on microfluidic chip for sequencing, we tested detecting EGFR multi-mutations on microfluidic chip. To mimic the real clinical samples in which EGFR-expressed cells account for a small portion and different types of mutations coexist in the same sample [35, 36], we mixed A549 cells (EGFR-expressed, wild type), NCI-H1975 cells (EGFR-expressed, point mutation L858R in exon 21 and T790M in exon 20), NCI-H1650 cells (EGFR-expressed, deletion mutation E746-A750 in exon 19) and HEK-293T cells (EGFR-unexpressed) at a ratio of 1:1:1:15. This ratio reflects a typical situation for tumor tissue in which EGFR-expressed cell account for about 10%–20% [35, 36]. A549, NCI-H1975 and NCI-H1650 cells are all NSCLC cells. L858R, T790M and E746-A750 are known as the most important mutations which are directly related to drug responses [1].

**Fig. 5:**
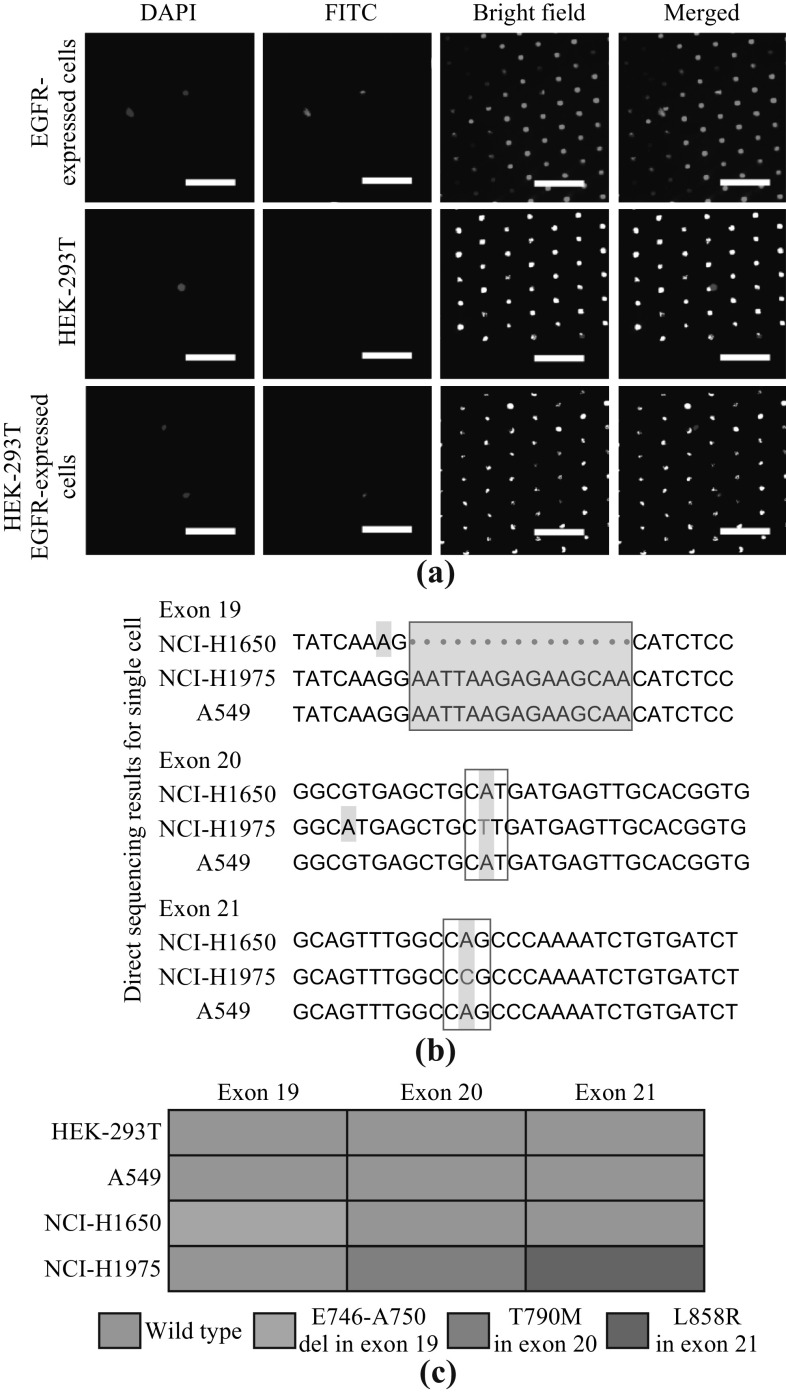
Detecting EGFR multi-mutations. **a** The fluorescent images of cells in microwells. Upper row is an area contains only EGFR-expressed cells which exhibit both DAPI (blue) and FITC (green) staining; middle row contains only HEK-293T cells which exhibit only DAPI (blue) staining; lower row contains both HEK-293T cells and EGFR-expressed cells. Scale bar: 100 µm. **b** The Sanger’s sequencing provides accurate mutation information: In exon 19, NCI-H1650 cells show a deletion mutation (Del E746-A750); in exon 20, NCI-H1975 cells show a point mutation (T790M); in exon 21, NCI-H1975 cells show a point mutation (L858R). **c** For NCI-H1975 cells, mutations in both exon 20 and 21 are detected; for NCI-H1650 cells, mutation in exon 19 is also detected. In addition, no false-positive result on either HEK-293T or A549 cells is detected. (Color figure online)

As shown in Fig. 5a, by utilizing anti-EGFR-FITC, the EGFR-expressed cells, including A549, NCI-H1975 and NCI-H1650 cells, were fluorescently identified and lysed. The cell lysates were collected for MDA amplification. To ensure precise sequencing results, we carefully determined the product quality of the first MDA amplification (as shown in Fig. S3). The DNA concentration, DNA mass and DNA fragment length all fully fulfilled the demands of sequencing multiple domains. The MDA amplification products were then divided into 3 parts and, respectively, amplified by PCR with 3 different primers for 3 domains of the EGFR gene (exon 19, 20 and 21). Figure 5b shows the results of direct sequencing (Sanger’s sequence results were shown in Fig. S4). It demonstrated that all mutated sequences were precisely detected, as long as one lysate collecting chamber contained lysate from < 4 cells. For comparison, directly sequencing the same mixture of A549, NCI-H1975, NCI-H1650, and HEK-293T cells (1:1:1:15) could not identify any EGFR mutations (Fig. S5). Figure 5c shows the statistic results of mutation analysis. It demonstrated that all mutations on both NCI-H1650 (Del E746-A750) and NCI-H1975 (T790M and L858R) cells were precisely detected, even under the circumstance that NCI-H1650 and NCI-H1975 cells account for a small portion of the whole cell population. Meanwhile, no false-positive result was found on either HEK-293T cells (EGFR-unexpressed) or A549 cells (no EGFR mutation), excluding potential misleading while performing targeted therapy. More importantly, in addition to detecting if specific mutations occurred, which could also be finished by expensive tissue-based NGS or ARMS (amplification refractory mutation system), our method also provided more single-cell-level information, on which specific cells the mutations occurred, or in another word, if any different mutations co-occurred on the same cells, or respectively occurred on different cells.

## Conclusion

Accurately discovering specific EGFR mutations, especially uncovering the mutation information from a small amount of mutated cells, which could be covered by the noises from other un-mutated cells, is currently becoming an urgent clinical requirement, since several key mutations have proven playing critical roles influencing drug responses of targeted cancer therapies. This requirement is yet to be satisfied with a simple, accurate and cost-effective method. This study provides a microfluidic-chip-based strategy in which the fluorescent identification of EGFR-expressed cells, in situ cell lysis, MDA and PCR gene amplification are integrated to provide high-quality gene amplification products from which the EGFR multi-mutations information could be acquired using simple and low-cost Sanger’s sequencing. This new strategy has the following prominent features: (1) by excluding cells without EGFR expression and limiting the cell numbers of each sequencing to < 4, or even only one cells, the majority of noises which interfere gene sequencing are excluded; therefore, the multi-mutations of a small portion of cells can be detected by simple and cheap Sanger’s sequencing, not expensive deep sequencing; (2) differs from expensive tissue-level NGS or ARMS method which are capable of detecting only the existence of specific mutations, our method provides other valuable single-cell-level information: on which specific cells the mutations occurred, or whether different mutations coexist on the same cells; (3) trapping and lysing single cells in microwells which are isolated from each other eliminate the cross-contamination and cell loss. Also, the combination of MDA and PCR amplification ensures the high quality of gene amplification products for acquiring accurate sequencing results. After optimizing the operation parameters, we verified the new strategy with cell mimics, which contain three most important EGFR mutations. The results reveal that the new strategy is capable of provide the answers of not only if the EGFR expression exists (by fluorescent identification), but also what the mutated sequences exactly are and on which cells these mutations occur.

Overall, for many clinical practices in which EGFR-expressed cells account for a small portion of the whole cell population, this study provides a new method for accurately detecting disease-related EGFR multi-mutations by employing a simple microfluidic chip and the cost-effective Sanger’s sequencing, as an economically affordable alternative to the expensive NGS or ARMS analysis of the whole cell population.

## Electronic supplementary material

Below is the link to the electronic supplementary material.

Supplementary material 1 (PDF 859 kb)
