# Supplementary material for: Identifying EGFR-Expressed Cells and Detecting EGFR Multi-Mutations at Single-Cell Level by Microfluidic Chip
Source: Nanomicro Lett. 2017 Nov 14;10(1):16. doi: 10.1007/s40820-017-0168-y (PMC6199061; doi:10.1007/s40820-017-0168-y)
Supplement: Supplementary file 1 — Supplementary material 1 (PDF 859 kb) [file 40820_2017_168_MOESM1_ESM.pdf]

Supplementary materials

Identifying EGFR-Expressed Cells and Detecting EGFR Multi-Mutations at Single-Cell Level by Microfluidic Chip

Ren Li[1, 2, 5, ‡], Mingxing Zhou[1, ‡], Jine Li[1], Zihua Wang[1], Weikai Zhang[1], Chunyan Yue[1], Yan Ma[1], Hailin Peng[2], Zewen Wei[1, *], Zhiyuan Hu[1, 3, 4, *]

[1]CAS Key Laboratory of Standardization and Measurement for Nanotechnology, CAS Key Laboratory for Biomedical Effects of Nanomaterials and Nanosafety, CAS Center for Excellence in Nanoscience， National Center for Nanoscience and Technology of China, Beijing 100190, People's Republic of China

[2]Academy for Advanced Interdisciplinary Studies, Peking University, Beijing 100871, People's Republic of China

[3]Sino-Danish College, University of Chinese Academy of Sciences, Beijing 100049, People's Republic of China

[4]Yangtze River Delta Academy of Nanotechnology and Industry Development Research, Jiaxing, Zhejiang Province, 314000, People's Republic of China

[5]University of Chinese Academy of Sciences, Beijing 100049, People's Republic of China

[‡]R. Li and M. Zhou contributed equally to the work

*Corresponding authors. E-mail: Huzy@nanoctr.cn, weizw@nanoctr.cn

Fax : 86-10-82545643 or 86-10-82545752

Figures

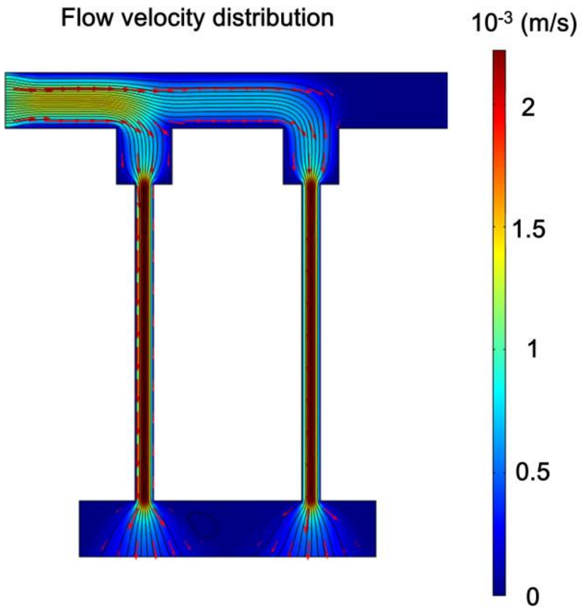

Fig. S1 The simulation of the flow velocity distribution in microfluidic chip

To study the mechanism of collecting the cell lysates from micro-wells, the flow velocity distribution in the microfluidic was analysed using FEA (Finite Element Analysis) software Comsol Ver. 5.0. The model dimensions were exactly the same with the real chip. The height of microfluidic channel was 30 μm, the side length of upper micro-well was 25 μm and the depth of upper micro-well was 30 μm. The width and depth of the through-hole were 8 and 170 μm, respectively. The flow velocity at the input was set to 15 μL min^{-1}. The simulation results suggest that no cross-contamination occurred between wells.

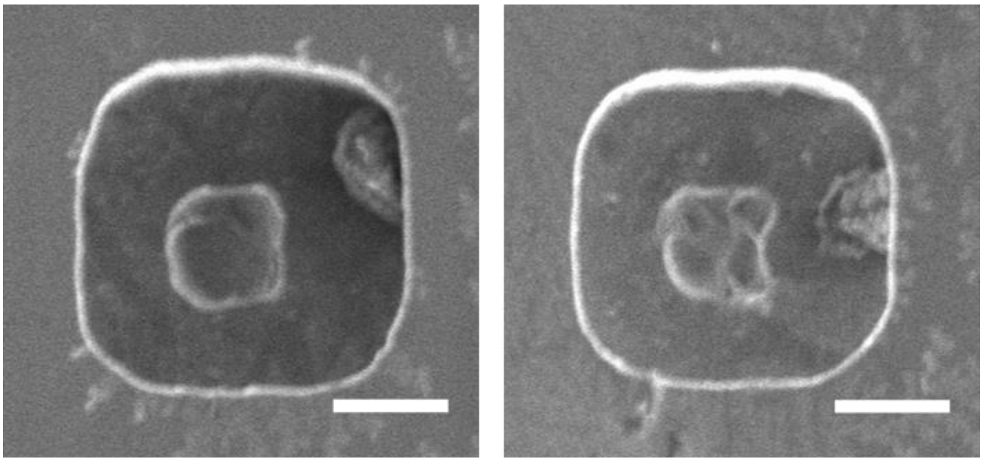

Fig. S2 Twin cells in one well

While the side-length of square micro-well is larger than 30 μm, one micro-well is tend to be occupied by two or more cells which is unacceptable for precise single cell fluorescent identification. Scale bar: 10 μm.

Sample	1	2	3	4
DNA concentration	192 ng/μL	181 ng/μL	220 ng/μL	216 ng/μL
DNA mass	9600 ng	9050 ng	11000 ng	10800 ng

(a)

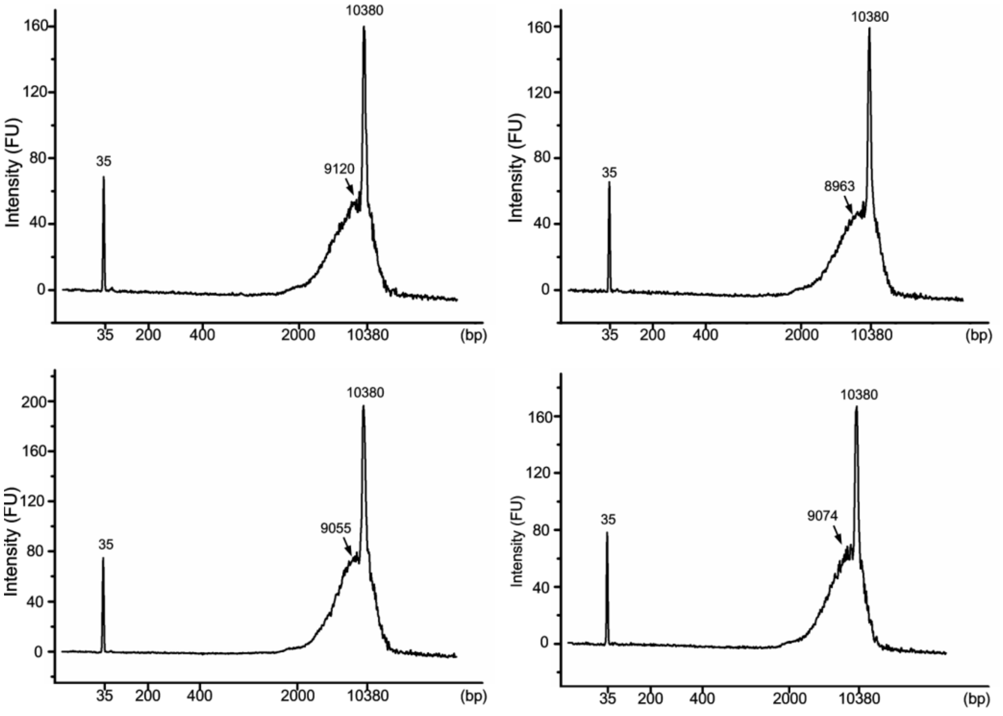

(b)

Fig. S3 The assessments of DNA amplification qualities

After amplifying cell lysates retrieved from separate cell lysate collecting chambers by MDA (Multiple Displacement Amplification). The amplification products were thoroughly examined to ensure optimum sequencing results. Theoretically, MDA amplifies the whole genome. We employed Qubit 3.0 fluorescence ration instrument (Thermo, USA) and Agilent 2100 bio-analyzer (Agilent, USA) to analysis the MDA products and DNA fragments length. For 4 different amplified samples, the DNA concentrations were 192, 181, 220, and 216 ng μL^{-1}, respectively. The total DNA mass in 4 samples were 9600, 9050, 11,000, and 10,800 ng, respectively. The fragments length main peak values were 9120, 8963, 9055, and 9074 bp, respectively. According to established standards[1], these parameters could more or less satisfy the requirements for the whole genome sequencing. Hence, the quality of MDA products would perfectly fulfil the demands of secondary amplifying and sequencing multiple specific gene domains.

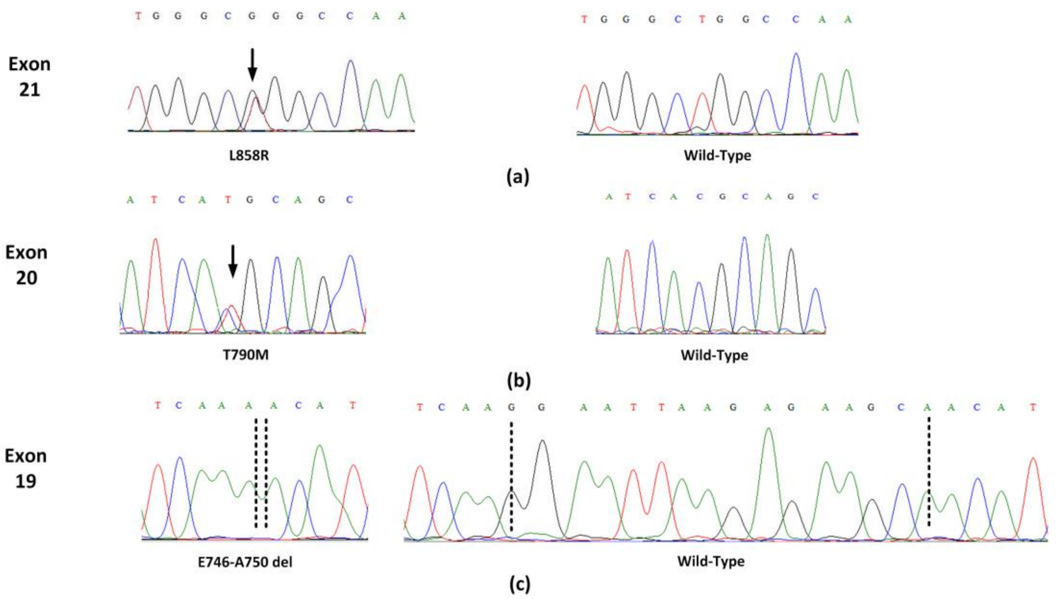

Fig. S4 The electropherograms of Sanger's gene sequencing (On chip processed)

The Supplementary Fig. S4 shows the electropherograms of Sanger's gene sequencing in exon 19, 20, and 21 of single cells. **a** The electropherograms of L858R mutation and wild type in exon 21. **b** The electropherograms of T790M mutation and wild type in exon 20. **c** The electropherograms of del E746-A750 mutation and wild type in exon 19. The arrows point mutation bases, and dashed lines show deletion bases. The wild-type indicates the homozygote which T790M and L858R mutation indicates the heterozygote.

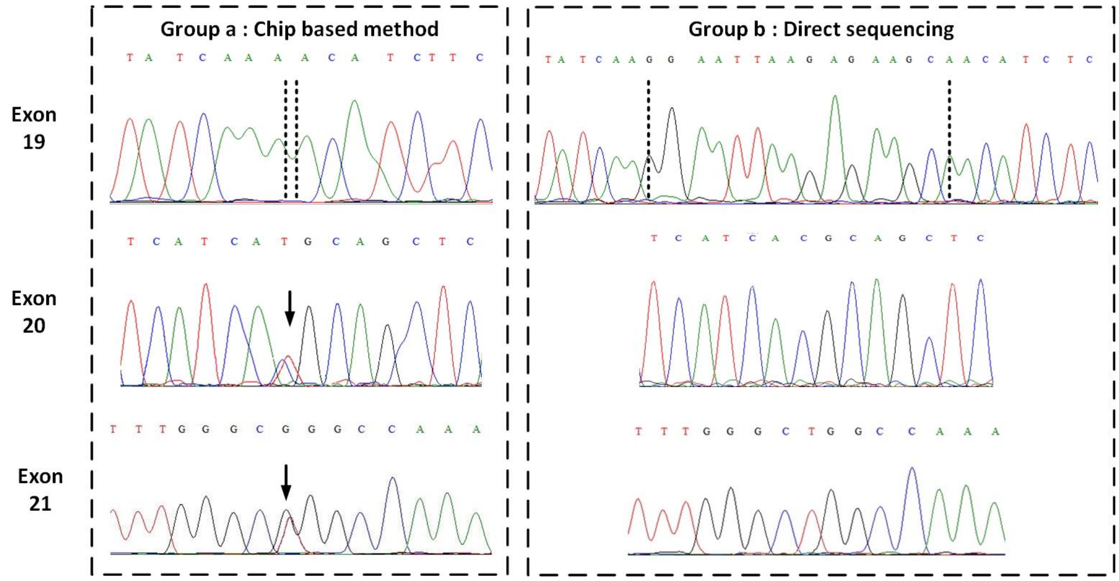

Fig. S5 The comparison of treating the same cell mixture with chip-based method and directly sequencing

A549 cells (EGFR-expressing, wild type), NCI-H1975 cells (EGFR-expressing, point mutation L858R in exon 21 and T790M in exon 20), NCI-H1650 cells (EGFR-expressing, deletion mutation E746-A750 in exon 19) and HEK-293T cells (without EGFR expression) were mixed at a ratio of 1:1:1:15. The cell mixture was equally divided into two groups: group **a**: cells were treated by chip-based method, including cell sorting, lysis, MDA amplification, PCR and Sanger' sequencing; group **b**: After performing cell lysis (by RIPA kit, Beyotime, China). The cell lysates were equally divided into 3 parts. PCRs were respectively performed on 3 parts with different primers:

Exon 19 forward: 5'-AACGTCTTCCTTCTCTCTCTGTCAT-3'

Exon 19 reverse: 5'-CACACAGCAAAGCAGAAACTCAC-3'

Exon 20 forward:
5'-ACCATGCGAAGCCACACTGACGTGCCTCTCCCTCCCTCCAG-3'

Exon 20 reverse:
5'-GTAATCAGGGAAGGGAGATACGGGGAGGGGAGATAAGGAGCCA-3'

Exon 21 forward: 5'-CCCTCACAGCAGGGTCTT-3'

Exon 21 reverse: 5'-GTCTGACCTAAAGCCACCTC-3'

Then the PCR products were sequenced by Sanger's method. All sequencing results of group (b) exhibited a wild type sequence while chip-based method provided correct sequences for all 3 kinds of mutations. It demonstrated that the mutation information from a minority of mutated cells was covered by other un-mutated cells, and chip-based method helped discovering the mutation information from the mutated cells.

SI Reference

[1] F.B. Dean, S. Hosono, L. Fang, X. Wu, A.F. Faruqi, et al., Comprehensive human genome amplification using multiple displacement amplification. Proc. Natl. Acad. Sci. **99**(8), 5261-5266 (2002). doi:10.1073/pnas.082089499
